# Usefulness of 3-Dimensional Computed Tomography Assessment of Femoral Tunnel after Anterior Cruciate Ligament Reconstruction

**DOI:** 10.3390/medicina59101716

**Published:** 2023-09-26

**Authors:** Min-Jeong Kim, Sung-Gyu Moon, Ji-Hee Kang, Dhong-Won Lee

**Affiliations:** 1 Department of Radiology, Incheon Sarang Hospital, Incheon 22135, Republic of Korea; ish1283@saranghospital.com; 2 Department of Radiology, KonKuk University Medical Center, Konkuk University School of Medicine, Seoul 05030, Republic of Korea; sgmoon@kuh.ac.kr (S.-G.M.); 20200184@kuh.ac.kr (J.-H.K.); 3 Department of Orthopaedic Surgery, KonKuk University Medical Center, Konkuk University School of Medicine, Seoul 05030, Republic of Korea

**Keywords:** anterior cruciate ligament anatomy, anterior cruciate ligament reconstruction, 3-dimensional computed tomography, tunnel position, femoral tunnel, quadrant method

## Abstract

Positioning of the femoral tunnel during anterior cruciate ligament (ACL) reconstruction is the most crucial factor for successful procedure. Owing to the inter-individual variability in the intra-articular anatomy, it can be challenging to obtain precise tunnel placement and ensure consistent results. Currently, the three-dimensional (3D) reconstruction of computed tomography (CT) scans is considered the best method for determining whether femoral tunnels are positioned correctly. Postoperative 3D-CT feedback can improve the accuracy of femoral tunnel placement. Precise tunnel formation obtained through feedback has a positive effect on graft maturation, graft failure, and clinical outcomes after surgery. However, even if femoral tunnel placement on 3D CT is appropriate, we should recognize that acute graft bending negatively affects surgical results. This review aimed to discuss the implementation of 3D-CT evaluation for predicting postoperative outcomes following ACL re-construction. Reviewing research that has performed 3D CT evaluations after ACL reconstruction can provide clinically significant evidence of the formation of ideal tunnels following anatomic ACL reconstruction.

## 1. Introduction

An anterior cruciate ligament (ACL) injury is a common sports-related knee injury that can cause knee instability and secondary meniscal damage [1]. The goal of ACL reconstruction is to restore the knee to a state that is anatomically, biomechanically, and functionally similar to normal [1]. Several factors are involved in achieving the best surgical outcomes, including the choice of graft, method of graft fixation, and postoperative rehabilitation exercises [2]. However, the positioning of the femoral and tibial tunnels during ACL reconstruction is the most crucial factor for successful procedure [3,4,5,6,7]. In particular, small changes in the femoral tunnel position can have large effects on the graft-length pattern during knee flexion and extension [8,9]. In other words, this can have significant implications not only for graft maturation and failure but also for knee biomechanics. Much research has been conducted on tunnel positioning during ACL reconstruction, and surgical techniques have evolved based on the evidence for establishing the position of the femoral tunnel [10,11,12,13,14,15,16,17,18].

Owing to inter-individual variability in the intra-articular anatomy, such as the bony ridges of the femoral condyle, it can be challenging to obtain precise tunnel placement and ensure consistent results, even when guided by intra-articular landmarks during reconstruction surgery. This concept of anatomical ligament reconstruction has led to an increase in the use of imaging to validate tunnel placement during and after surgery [10,11,12,13,14,15,16,17,18,19,20,21]. A consensus on the anatomical centers of the femur and tibia is crucial for practical and clinically efficient use of imaging to confirm tunnel placement [22].

The accuracy of imaging in depicting tunnel placement has been validated in cadaver studies using radiographs, computed tomography (CT) scan, and magnetic resonance imaging (MRI) [23]. Currently, three-dimensional (3D) reconstruction of CT scans is considered the best method to determine whether the femoral and tibial tunnels are positioned correctly [23,24].

This narrative review aims to present the formation of ideal femoral tunnels and to discuss the implementation of 3D-CT evaluation for predicting postoperative outcomes following ACL reconstruction based on the current literature.

## 2. Femoral Tunnel Assessments

### 2.1. Cadaveric Anatomy

**Figure 1:**
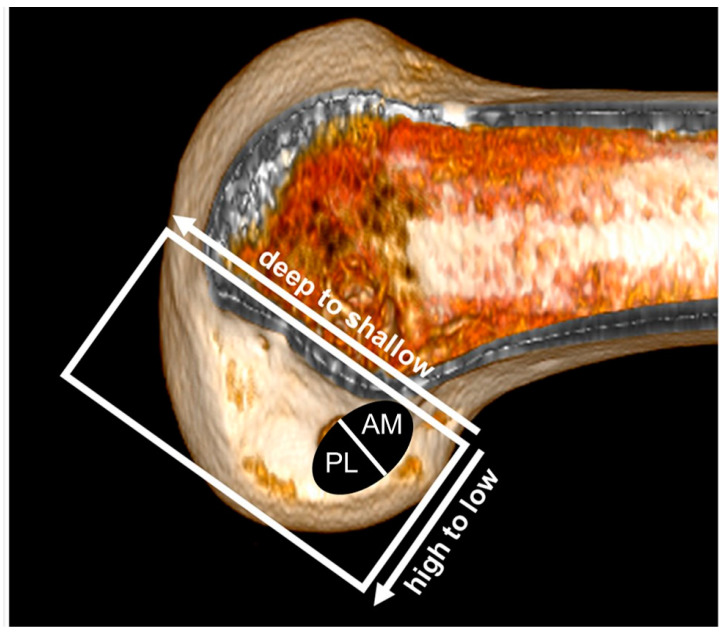
Femoral tunnel placement by the quadrant method. AM—anteromedial; PL—posterolateral.

Several studies assessed the femoral footprint of the native ACL in cadaveric specimens [25,26,27,28,29,30,31]. These studies used the Bernard and Hertel grid, known as the quadrant method, as described by Bernard et al. [32] to represent the ACL center. The center of the ACL footprint was determined in the high to low direction and deep to shallow direction and presented as the percentage of the distance from the roof of the intercondylar notch and the posterior edge of the lateral femoral condyle (Figure 1). The centers of anteromedial (AM) bundle and posterolateral (PL) bundles are summarized in Table 1.

Piefer et al. [33] systematically reviewed the anatomic center of the ACL femoral footprint. After analyzing eight items, the mean whole bundle center was 35.2% for the high to low direction and 28.5% for the deep to shallow direction. The mean centers of the AM bunle and PL bundle were 23.1% and 48.8% in the high to low direction and 21.5% and 32% in the deep to shallow direction, respectively. Parkar et al. [22] conducted a systematic review of the literature on the anatomic center of the ACL. In their systematic review, 218 knees demonstrated that the weighted mean and the weighted median of the femoral center were 35% and 34% for the high to low direction and 29% and 26% for the deep to shallow direction, respectively. The weighted 5th and 95th percentiles for high to low direction were 28% and 43%, and for deep to shallow direction were 24% and 37%, respectively. They suggested the use of the 5th and 95th percentiles when evaluating postoperative femoral placement to be ‘in or out of the anatomic range.’ These systematic reviews of anatomy studies have clinical relevance for orthopedic surgeons who believe that anatomical ACL reconstruction results in improved outcomes.

### 2.2. Postoperative 3D CT-Based Study

Intra-articular anatomy is influenced by inter-individual variability, and in anatomical studies, structures such as the lateral intercondylar ridge or bifurcate ridge of the femoral condyle have been described to exhibit variations among individuals [34,35,36]. Moreover, in cases of chronic rupture or re-rupture, these intra-articular structures are often not clearly visible, making it challenging for surgeons to determine whether the femoral tunnel has been accurately positioned during the operation [37]. Therefore, using only intra-articular landmarks may result in significant variations in femoral tunnel placement. To overcome this problem, many authors are trying to use intraoperative fluoroscopy or ACL computer-assisted surgery, but this is still at a challenging stage, and further verification studies are needed [38,39].

**Figure 2:**
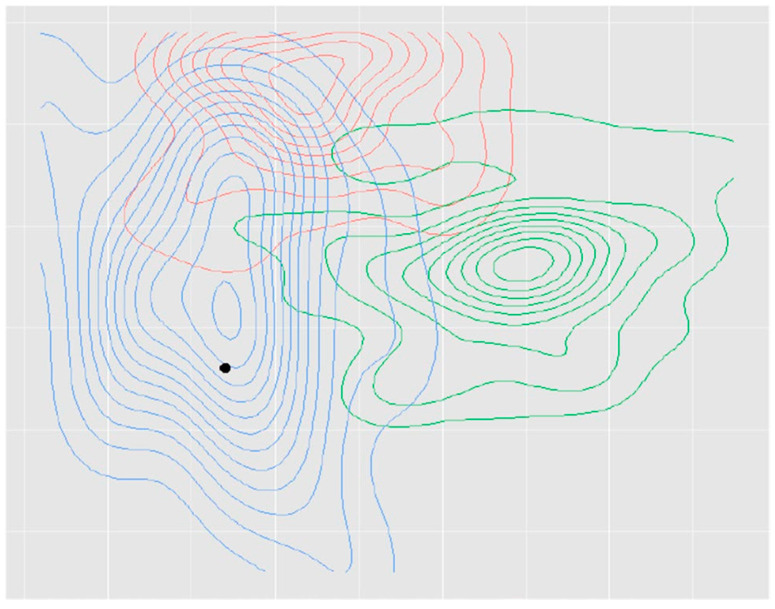
Contour plot of the spread of the femoral tunnels in transtibial group, trans-AM portal technique without feedback (AM 1) and trans-AM portal technique after feedback (AM 2) groups (red transtibial group, green AM 1 group, blue AM 2 group). Black dot means an ideal femoral tunnel center (34% in high to low direction and 27% in deep to shallow direction) described by Bird et al. [19]. (Reprinted with permission from Springer [38]).

**Figure 3:**
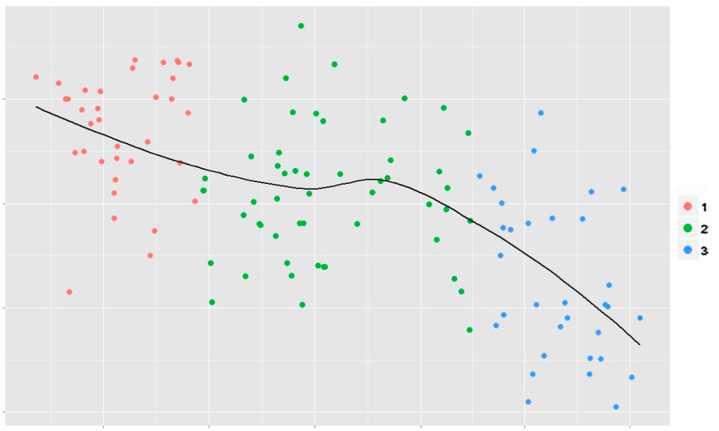
Relative distance (*Y*-axis indicates relative distance from ideal femoral tunnel center in absolute value) from femoral tunnel to ideal femoral tunnel center as a function of time (*X*-axis indicates time from first to last surgery). *Red (1) transtibial group, green (2) trans-AM portal technique without feedback, blue (3) trans-AM portal technique after feedback*. (Reprinted with permission from Springer [38]).

The usefulness of feedback after ACL reconstruction using 3D-CT has been reported in several studies [10,38,40]. Inderhaug et al. [38] switched from a transtibial to a trans-AM portal technique for ACL reconstruction, and they compared the femoral tunnel positions between the trans-AM portal technique without postoperative 3D-CT feedback (AM 1) and the trans-AM portal technique after 3D-CT feedback (AM 2). Mean femoral tunnel placements in high to low direction were 8%, 26%, and 29% in transtibial group, AM 1, and AM 2, and for deep to shallow direction were 32%, 43%, and 27% in transtibial group, AM 1, and AM 2, respectively. The femoral tunnel placement gradually approached the ideal femoral tunnel center (34% in high to low direction and 27% in deep to shallow direction) described by Bird et al. [19] in all groups over time. In particular, AM 2 showed a significant improvement in the distance from the ideal femoral tunnel center over time throughout the feedback period compared to AMI 1 (*p* = 0.001) (Figure 2). Changing from the transtibial to the trans-AM portal technique and postoperative 3D-CT feedback were critical points in the improvement of femoral tunnel placement (Figure 2 and Figure 3). It is important to perform repeated surgeries to increase the accuracy of femoral tunnel placement; however, radiological feedback is equally important. Sirleo et al. [10] performed ACL reconstruction using the outside-in technique in a series of 60 consecutive patients, providing 3D-CT feedback within 48 h after each surgery. Subsequently, they divided the patients into three groups of 20 patients each according to the order of surgery (three consecutive series) and compared the femoral tunnel placement among these groups. The ideal femoral tunnel placement was 35.2% in high to low direction and 28.5% in deep to shallow direction, reported by Piefer et al. [33]. They confirmed progressive improvement in femoral tunnel placement from the first to the second series, and from the second to the third series. Both the accuracy and precision of the femoral tunnel placement increased from the first to the third series. They concluded that postoperative 3D-CT feedback was effective in the learning process to improve accuracy (+52.4%) and precision (+55.7%) of femoral tunnel placement to perform appropriate anatomic ACL reconstruction.

### 2.3. Clinical Relevance of Postoperative 3D CT

Parkinson et al. [41] demonstrated that shallow nonanatomic femoral tunnel placement was significant predictor of graft failure (hazard ratio 4.3; 95% CI, 1.6–11.6; *p* = 0.004). Among 97 patients assessed using postoperative 3D-CT, failure rates were 30% for shallow nonanatomic placement and 21% for deep anatomic placement. Shallow femoral tunnel placement is the most common cause of ACL reconstruction failure [42,43,44]. They recommended postoperative 3D-CT to ensure appropriate femoral tunnel placement and to decrease the risk of non-anatomic femoral tunnel positioning and failure. Mhaskar et al. [40] analyzed 60 ACL reconstructions and categorized femoral tunnel placement into type I (well-placed), type II (slightly malpositioned), and type III (grossly malpositioned) according to postoperative 3D-Ct. There were 32 type I, 28 type II, and no type III tunnels. There were significant differences in postoperative Lysholm score and International Knee Documentation Committee (IKDC) score between type I and II tunnels (62.2 ± 16.2 vs. 48.5 ± 17.2, *p* = 0.002, and 62.5 ± 14.3 vs. 52.7 ± 15.1, *p* = 0.012, respectively).

Some authors reported that femoral tunnel placement on 3D-CT can influence graft inclination and maturation on MRI. Lee et al. [45] reported that a total of 60 patients who underwent anatomical ACL reconstruction using flexible reamer system showed femoral tunnel located at 24.1 ± 5.9% in high to low direction and at mean 29.7 ± 4.4% in deep to shallow direction on postoperative 3D-CT. They aimed to target the femoral tunnel position slightly toward the AM bundle rather than the center of the entire bundle and received 3D-CT feedback after each procedure. As a result, all patients had a comparable sagittal and coronal graft inclination to them of the native ACL (52.4 ± 4.6° and 69.2 ± 4.7°, respectively). Femoral tunnels created at anatomical positions on 3D-CT ultimately result in a graft inclination similar to that of the normal ACL, and this similarity in graft inclination can have implications for clinical outcomes and graft laxity [46,47]. Lee et al. [48] proved that positioning the femoral tunnel near the AM bundle and center led to better graft signal intensity on postoperative MRI (one year after anatomic ACL reconstruction) than positioning the femoral tunnel near the PL bundle, although there were no differences in clinical outcomes or knee laxity. MRI signal intensity reflects graft maturation [16,48,49]. In their study, the mean femoral tunnel placements were 20.3%, 33.76%, and 47.56% in high to low direction in AM bundle positioned group, center positioned group, and PL bundle positioned groups, respectively. Regarding deep to shallow direction, mean femoral tunnel placements were 21.67%, 25.95%, and 30.23% in AM bundle positioned group, center positioned group, and PL bundle positioned groups, respectively. They assumed that the AM bundle and center positions led to less graft excursion during knee flexion and extension than the PL bundle position.

Even when similar femoral tunnel positions are observed on 3D-CT, the graft bending angle varies between femoral drilling techniques. Because the graft bending angle can influence graft stress, it is necessary in the future to analyze not only the tunnel position but also the graft bending angle for receiving feedback. Lee et al. [16] compared the femoral tunnel placement, the graft bending angle at the femoral tunnel on 3D-CT, and the signal/noise quotient (SNQ) on MRI between single-bundle ACL reconstruction using modified transtibial and outside-in techniques. Femoral tunnel placement for the high to low direction were 39.8 ± 2.4% and 40.9 ± 1.9% (*p* = 0.087), and deep to shallow direction were 31.3 ± 2.9% and 33.2 ± 2.5% (*p* = 0.517) in modified transtibial group and outside-in group, respectively. However, the femoral graft bending angle was reduced in the modified transtibial group, and SNQ at the femoral intraosseous and proximal grafts on MRI were lower in the modified transtibial group than in the outside-in group (*p* < 0.01). These results showed that an acute femoral graft bending angle could negatively affect graft maturation, even though the femoral tunnel was positioned anatomically. Niki et al. [21] compared the femoral tunnel placement and graft bending angle on 3D-CT between double-bundle ACL reconstruction using outside-in and trans-AM portal techniques. There were no significant differences in femoral tunnel placement between the two groups. Femoral tunnel placement for the high to low direction were 19.8 ± 6.2% (AM bundle) and 47.9 ± 8.5% (PL bundle) in the outside-in group, and 22.9 ± 8.0% (AM bundle) and 48.6 ± 8.8% (PL bundle) in the trans-AM portal group. The deep-to-shallow directions were 18.4 ± 5.6% (AM bundle) and 22.5 ± 6.0% (PL bundle) in the outside-in group, and 21.0 ± 5.5% (AM bundle) and 24.6 ± 4.8% (PL bundle) in the trans-AM portal group. However, the graft bending angles of the AM bundle and PL bundle were greater in the outside-in group (*p* < 0.001). These results can help surgeons choose a technique that shows an obtuse graft bending angle while creating a femoral tunnel in an appropriate position.

The 3D-CT can provide surgeons with useful information for revision ACL reconstruction. During revision ACL reconstruction, accurate determination of the position of the femoral tunnel created is essential for planning whether revision surgery should be performed in a one- or two-stage manner. In this context, 3D-CT serves as a tool to accurately assess femoral tunnel placement. Magnussen et al. [50] developed a femoral tunnel classification according to 3D-CT, which yielded a moderate to substantial inter- and intra-observer reliability (kappa coefficient of 0.57 and 0.67, respectively) (Table 2). They suggested that type I should consider a re-using tunnel, type 2 should consider a two-stage procedure when there is a possibility of tunnel convergence, and type 3 should consider a new tunnel without concern of convergence.

## 3. Authors’ Suggestions

Because it is important to create a femoral tunnel at the anatomical footprint for successful ACL reconstruction, surgeons must accurately identify the anatomical femoral footprint as identified in various cadaveric experiments. However, owing to the large individual variations in bony landmarks, it is often difficult to evaluate whether the femoral tunnel is created in the desired position when operated in a limited arthroscopic field of view. If a surgeon does not recognize that the femoral tunnel placement is incorrect and performs the same surgery, the surgeon is likely to make the same mistake. To avoid such errors, radiological feedback is required. In order to receive feedback on the target point you chose during surgery, it is better to review it within 48 to 72 h after the surgery.

In addition, it is important for surgeons to know the results of the surgery and explain them to the patient.

## 4. Conclusions

Postoperative 3D-CT feedback, which is considered the best method for determining whether femoral tunnels are positioned correctly, is effective in improving the accuracy of femoral tunnel placement. Precise tunnel formation obtained through feedback may have a positive effect on graft maturation, graft failure, and clinical outcomes after surgery.

## 5. Future Directions

Even if femoral tunnel placement on 3D-CT is appropriate, we should recognize that acute graft bending negatively affects surgical results. Therefore, in the future, it will be necessary to utilize 3D-CT to analyze femoral tunnel placement as well as various factors such as the graft bending angle and graft contact area. This will enable the receipt of feedback that will facilitate the selection of appropriate surgical techniques.

## Figures and Tables

**Table 1:** Anatomic Center of ACL Femoral Footprint.

Study	Whole Bundle		Anteromedial Bundle		Posterolateral Bundle	
	High to Low (%)	Deep to Shallow (%)	High to Low (%)	Deep to Shallow (%)	High to Low (%)	Deep to Shallow (%)
Colombet et al. [25]	36.5	29.4	25.3	26.4	47.6	32.3
Forsythe et al. [31]	44.3	28.4	33.2	21.7	55.3	35.1
Lee et al. [26]	41.0	37.3	25.6	33.9	56.4	40.6
Lorenz et al. [29]	33.5	24.0	22.0	21.0	45.0	27.0
Luites et al. [27]	28.5	25.5	10.0	23.0	47.0	28.0
Musahl et al. [30]	26.6	26.3	Not applicable	Not applicable	Not applicable	Not applicable
Tsukada et al. [28]	30.0	30.4	17.8	25.9	41.1	34.8

**Table 2:** Classification system for previous tunnel placement according to 3D-CT.

Femoral Tunnel Type		Location Relative to the Lateral Intercondylar Ridge
I	Well positioned	Inferior and posterior
II-Vertical	Slightly malpositioned	Overlapping
II-Anterior		
II-Both		
III	Significantly malpositioned	Entirely vertical and/or anterior

## Data Availability

All relevant data generated or analyzed during this study are included in this published article.
